# Non-viral targeted integration at the CISH locus enables CAR-NK cell engineering with enhanced anti-tumor activity

**DOI:** 10.1016/j.omton.2026.201206

**Published:** 2026-04-17

**Authors:** Jiao Wang, Yao Sun, Jakob Starzyk, Fei Wang, Xin Dong, Richard Shan, Xuemei He, Keqiang Xie, Guozhu Xie, Hao Wu

**Affiliations:** 1 Full Circles Therapeutics, Inc. 625 Mt. Auburn St., Ste. 105, Cambridge, MA 02138, USA; 2 Department of Radiation Oncology, Nanfang Hospital, Southern Medical University, Guangzhou 510515, China; 3 The First School of Clinical Medicine, Southern Medical University, Guangzhou 510515, China

**Keywords:** MT: Regular Issue, CLICK, circular single-stranded DNA, non-viral CAR NK, allogeneic cell therapy, CISH

## Abstract

Natural killer (NK) cells hold promise for adoptive cell therapy due to their innate cytotoxicity. Early clinical trials confirm their safety and efficacy in cancer and autoimmune disease treatment. Engineering NK cells with chimeric antigen receptors (CARs) enhances target specificity and facilitates their development as off-the-shelf allogeneic therapies. However, both viral and non-viral engineering methods of NK cells present challenges. Here, we introduce *CISH* locus integrated CAR killer (CLICK), a novel non-viral approach using a mini-circular single-stranded DNA genome editing system. CLICK enables efficient integration of CD19CAR sequences into the metabolic checkpoint *CISH* locus, simultaneously disrupting *CISH* and driving stable, progressively increasing CAR expression in peripheral blood-derived NK cells. CLICK-engineered CAR-NK cells exhibit potent cytotoxicity, enhanced anti-tumor activity *ex vivo* and *in vivo*, extended persistence, and reduced exhaustion. Together, these findings highlight CLICK as a highly efficient and versatile platform for non-viral CAR-NK cell engineering, offering a scalable approach for next-generation allogeneic immune cell therapies.

## Graphical abstract

**Figure:**
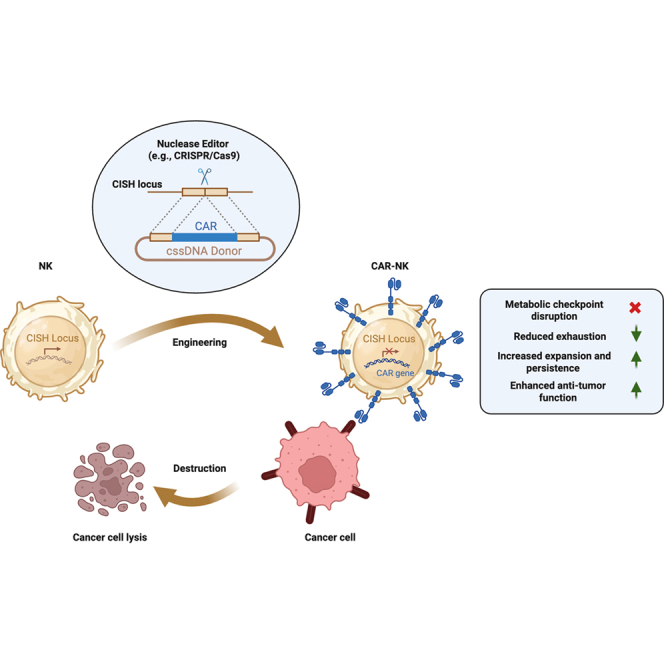

## Teaser

Wu and colleagues introduce CLICK, a non-viral strategy that integrates CARs into the *CISH* locus in NK cells, simultaneously disrupting a metabolic checkpoint and enabling sustained CAR expression. This dual mechanism enhances persistence and antitumor activity, supporting scalable production of next-generation allogeneic CAR-NK cell therapies.

## Introduction

The demand for allogeneic engineered immune cell therapies that are safe, effective, and affordable is rapidly increasing. Unlike T cells, NK cells can rapidly induce direct cytotoxicity without prior antigen priming.^1^^,^^2^^,^^3^^,^^4^^,^^5^^,^^6^^,^^7^^,^^8^ Key advantages of NK cell-mediated anti-tumor activity include (1) the use of multiple mechanisms for tumor recognition and (2) the absence of rearranged antigen receptors, which facilitates the development of “off-the-shelf” cellular products with improved safety profiles, including minimal risk of cytokine release syndrome, neurotoxicity, and graft-versus-host disease (GvHD).^2^^,^^3^^,^^4^^,^^7^^,^^9^

Despite these advantages, NK cell therapies face challenges such as poor persistence and reduced activity at tumor sites.^8^^,^^10^^,^^11^^,^^12^ Genetic engineering offers a promising strategy to enhance NK cell persistence and cytotoxicity.^13^^,^^14^^,^^15^^,^^16^ NK cells engineered with chimeric antigen receptors (CARs) have shown enhanced target specificity, demonstrating preclinical efficacy in both hematologic malignancies and solid tumors^8^^,^^16^^,^^17^^,^^18^ as well as clinical efficacy in hematologic cancers.^9^ However, NK cells exhibit resistance to exogenous DNA uptake, leading to low genome-editing efficiency.^19^^,^^20^ While lentiviral and retroviral vectors improve gene transfer efficiency, they present several challenges, including (1) inconsistent transduction due to the lack of viral receptors, (2) limited cargo size, (3) difficulties in large-scale manufacturing, and (4) safety concerns from random insertional mutagenesis.^15^^,^^21^^,^^22^^,^^23^ Therefore, a more robust, precise, and safer genetic toolkit for NK cell engineering is urgently needed.

Recent advancement in CRISPR-Cas9 gene editing technology has reinvigorated interest in genome editing of human NK and T cells.^8^^,^^16^^,^^17^^,^^18^^,^^24^^,^^25^^,^^26^ AAV6 has been widely used as a donor carrier for homology-directed repair (HDR)-mediated NK cell engineering, achieving high knock-in (KI) efficiency and enhanced anti-tumor activity.^8^^,^^11^^,^^17^ However, AAV6 poses safety concerns, manufacturing challenges, and limitations due to its 4.5 kb packaging constraint. Non-viral approaches, including double-stranded linear DNA (dsDNA) and single-stranded linear DNA (ssDNA) donor templates, have been explored but suffer from low efficiency and high cytotoxicity.^25^^,^^27^ Innovative strategies such as the sleeping beauty transposase-based non-viral NK cell engineering have recently demonstrated promising cytotoxic functions.^28^ Other studies have utilized Baboon envelope-pseudotyped lentiviral vectors for genetically engineered CAR-NK cells in oncology research.^29^^,^^30^ Additionally, more efficient non-viral NK cell engineering using circular single-stranded DNA (cssDNA) donor templates for targeted transgene integration with the CRISPR-Cas9 system has been reported.^25^

Here, we present a *CISH* locus integrated CAR killer (CLICK), a modular non-viral targeted genome integration platform that utilizes phagemid-derived cssDNA as an optimized donor vector for HDR-mediated CAR KI at the *CISH* locus. *CISH* encodes CIS protein, a metabolic checkpoint in NK and T cells, functions as a negative regulator of the JAK-STAT signaling pathway by binding to activated JAK kinases following cytokine stimulation (e.g., interleukin-2 [IL-2] or IL-15). This interaction inhibits further phosphorylation and activation of STAT transcription factors, serving as a molecular break to modulate the intensity and duration of the signaling response.^31^^,^^32^^,^^33^^,^^34^ Ablation of *CISH* expression has been applied as a strategy to enhance immune cell expansion, reduce exhaustion and demonstrated improved antitumor activity.^35^^,^^36^^,^^37^ Thus, *CISH* serves as an ideal target locus for precise, non-viral CAR-NK cell engineering, with simultaneous *CISH* ablation. Our studies demonstrate that CLICK enables efficient KI while minimizing DNA-mediated cytotoxicity in peripheral blood-derived primary NK cells. Specifically, we show that cssDNA donor templates can be co-delivered with Cas9-sgRNA ribonucleoprotein complexes (RNPs) to achieve targeted insertion of multi-kilobase coding sequences at the optimized *CISH* locus. This engineering strategy not only disrupts the metabolic checkpoint (*CISH*) but also enables targeted KI of multiple expression constructs, such as CD19CAR. CLICK-engineered CAR-NK cells exhibit locus-specific CAR expression enrichment during *ex vivo* expansion, extended persistence, and reduced exhaustion *in vivo*, maintaining durable anti-tumor functions.

The CLICK platform provides an efficient and modular method for non-viral engineering of functional CAR-NK cells, improving persistence and cytolytic function. This two-in-one KO/KI approach—simultaneously disrupting the CISH immune checkpoint and enabling targeted CAR KI—has the potential to expand the use of genetically engineered NK cells as off-the-shelf allogeneic therapies for cancer, autoimmune disorders, and other relevant disease indications.

## Results

### Human NK cells culture and expansion

Numerous protocols exist for the *ex vivo* expansion of primary NK cells. Here, we adapted a robust feeder cell-driven culture method that facilitates the highly efficient growth and activation of cryopreserved NK cells.^38^ Compared with feeder-free conditions, NK cells cultured in medium containing IL-21 and mitomycin C-pretreated feeder cells (Epstein-Barr virus-transformed lymphoblastoid cells, EBV-LCL) exhibited a significantly more rapid and sustained expansion. As shown in Figure S1A, traditional feeder-free cultures yielded a maximum ∼80-fold expansion 35 days post recovery. In contrast, NK cells cultured with feeder cells exhibited over 5000-fold expansion within the same period, with the potential for further amplification. Moreover, NK cells expanded in the feeder cell condition demonstrated enhanced cytolytic activity against K562 target cells compared to those expanded in feeder-free conditions (Figure S1B).

Cryopreservation of NK cells using freezing media containing 10% dimethyl sulfoxide (DMSO) has been reported to reduce post-thaw viability and function.^39^ To mitigate this effect, we implemented a modified two-phase expansion strategy for systematic optimization. Phase 1 (days 0–7) was designed to promote cell recovery post-thaw. During this phase, NK cells were thawed and maintained in a feeder-free medium (NK MACS with 500 U/mL IL-2). Phase 2 (from day 7 onward) aimed to maximize expansion potential and generate a high yield of activated NK cells. Feeder cell co-culture was initiated in this phase, and NK cell expansion and viability were monitored. As shown in Figures S1C and S1D, the introduction of Phase 1 resulted in a relatively faster and more sustained expansion. Additionally, the feeder cells were cleared more rapidly in the two-phase expansion setting (approximately day 10 vs. day 14 in standard conditions).

Furthermore, we conducted a comparison of the expansion efficiency of cryopreserved NK cells using our defined two-phase expansion protocol with the direct one-phase expansion for the freshly isolated primary NK cells from the same donor. Both fresh and cryopreserved NK cells exhibited rapid and sustained expansion in a comparable manner (Figures S1E and S1F). Collectively, these results support a recovery and expansion process that enables the generation of highly proliferative and activated NK cells. This optimized NK cell culture approach was subsequently applied to our initial genome editing study using the cssDNA non-viral engineering platform and screening campaign of sgRNAs.

### Non-viral transgene KI of primary NK cells with cssDNA HDR donor template

We previously demonstrated GFP reporter and CD19CAR transgene integration at the *RAB11A* locus using cssDNA-mediated genomic integration.^25^ In this study, we extended our evaluation to multiple donor NK cells, integrating a clinically validated CD19-1XX CAR construct^40^^,^^41^ into the *RAB11A* locus using the CRISPR-Cas9 and cssDNA engineering system (Figures S2A and S2B). Thereafter, we referred to CD19-1XX CAR as CD19CAR in this study. KI efficiencies of CD19CAR, assessed 7 days post-electroporation, varied between donors, with donor #1 achieving ∼20% efficiency and donor #2%–10% (Figures S2C–S2F). Despite this variability, both CD19CAR-NK populations exhibited the expected cytolytic function against CD19^+^ NALM6 B lymphoblastoid cells and maintained desirable cell viability and expansion profiles in culture (Figures S2G–S2L).

In addition, to assess freeze-thaw tolerance, we evaluated NK cells from donor #2. Immediately after thaw (day 0), cssDNA-engineered CD19CAR-NK cells maintained stable CD19CAR expression (Figures S2M and S2N). Although freeze-thaw reduced cytolytic activity across all groups, CD19CAR-NK cells consistently exhibited the highest cytotoxicity against NALM6 cells (Figure S2O). High post-thaw viability (>85%) was observed in all groups (Figures S2P–S2R). Following an additional 7 days of culture post-thaw, CD19CAR expression remained stable in cssDNA-engineered NK cells (Figures S2S and S2T). Cytotoxic function recovered across all groups, with CD19CAR-NK cells again demonstrating superior antitumor activity (Figure S2U). Cell viability remained high (>85%) across all groups (Figure S2V).

### Identification of genomic targeting site on *CISH* locus

The *CISH* gene has emerged as a promising therapeutic target for enhancing NK cell-mediated cancer immunotherapy. As a metabolic regulator signaling through the JAK/STAT pathway in the immune cells, *CISH* is considered a molecular break sensitizing the external interleukin stimulation required for immune cells fitness. Genetic ablation and inhibition of *CISH* in NK cells have been shown to stimulate mTOR signaling, improve NK cell metabolic fitness, and enhance NK cell persistence within the tumor microenvironment (TME), ultimately leading to improved antitumor immunity.^31^^,^^35^^,^^37^^,^^42^ Leveraging the capabilities of the cssDNA-mediated genome integration platform, along with the previously demonstrated results, we propose that this novel non-viral genome editing technology enables a “two-in-one” editing approach, simultaneously ablating *CISH* to enhance NK cell expansion and fitness while integrating functional CAR structures (e.g., CD19CAR) to direct NK cells against tumor cells with high target specificity.

**Figure 1:**
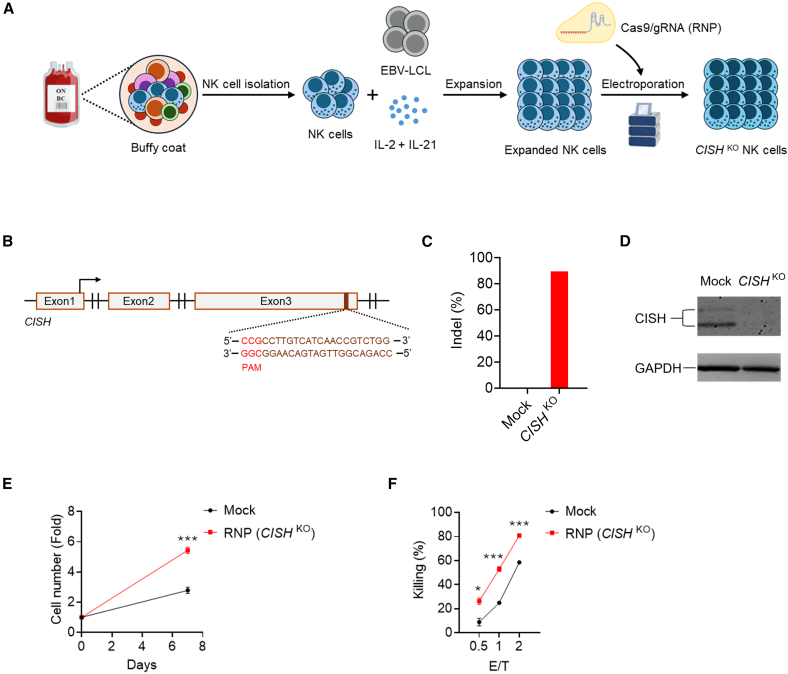
CRISPR-Cas9-mediated deletion of *CISH* in NK cells (A) Schematic diagram of *ex vivo* expansion and CRISPR-Cas9 gene editing approach for NK cell genetic engineering on *CISH* locus. NK cells were isolated from healthy donor PBMCs and co-cultured with EBV-LCL feeder cells plus IL-2 and IL-21 for around 2 weeks. Expanded NK cells were electroporated using precomplexed Cas9 and *CISH*-targeting sgRNAs. Edited NK cells were then expanded further in NK MACS media and analyzed for KO efficiency, proliferation, and function. (B) Schematic representation of CRISPR-Cas9-mediated CISH KO using the in-house screened sgRNA targeting exon 3 of the CISH gene. PAM, protospacer-adjacent motif. (C) Indel (%) at the *CISH* locus was measured by NGS amplicon sequencing. (D) Western blot assay for *CISH* protein expression in CRISPR-Cas9-engineered NK cells. GAPDH was used as loading control. (E) *In vitro* proliferation of mock NK cells and sgRNA-Cas9 RNP complex-mediated *CISH* knockout NK cells (*CISH*^KO^). Data represent mean ± SEM of 3 independent replicates. ∗∗∗*p* < 0.001. *p* values were determined using the two-tailed Student’s *t* test analysis. (F) *In vitro* cytotoxicity of mock NK cells and *CISH*^KO^ NK cells against K562 target cells co-cultured at various effector-to-target (E:T) ratios for 4 h. Data represent mean ± SEM of 3 independent replicates. ∗*p* < 0.05, ∗∗∗*p* < 0.001. *p* values were determined using the two-tailed Student’s *t* test analysis.

To test this hypothesis, we performed an sgRNA screening campaign and identified a specific locus within exon 3 of *CISH* with a high cutting efficiency (>90% indel) (Figures 1A–1C; Table S1; Figure S3A). Exon 3 encodes the C-terminal SOCS box domain, a critical ubiquitin-binding site that facilitates interactions between CISH and E3 ubiquitin ligases, targeting proteins for degradation via the ubiquitination pathway.^43^ Following genome engineering, CISH protein expression was barely detectable in Cas9 RNP-electroporated NK cells, confirming the highly effective on-target ablation achieved with the selected sgRNA (Figure 1D; Figure S3B). Moreover, *CISH* ^KO^ NK cells exhibited significantly improved *ex vivo* proliferation and enhanced cytotoxicity against K562 target cells (Figures 1E and 1F; Figures S3C and S3D).

### cssDNA HDR donor template mediated CD19CAR KI at functional *CISH* locus

**Figure 2:**
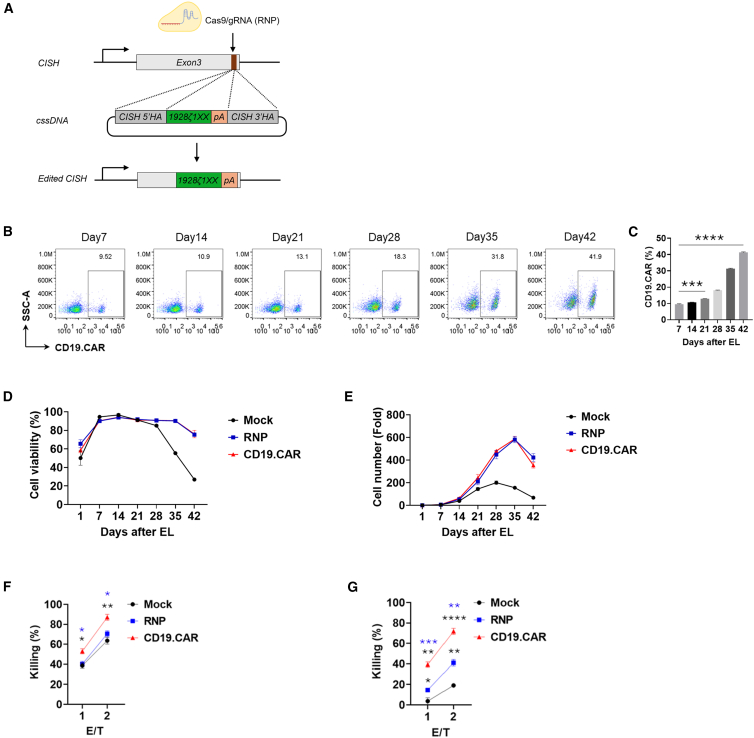
Efficient CD19CAR-NK engineering at *CISH* locus by CLICK (A) Schematic diagram of cssDNA donor template used for the genome insertion of CD19CAR (1928ζ1XX) transgene at *CISH* locus with the screened sgRNA targeting the 3′ end of exon 3 of *CISH* locus. (B and C) Representative flow cytometry profiles (B) and quantifications (C) of CD19CAR knock-in efficiency at *CISH* locus (CD19CAR %) on day 7, 14, 21, 28, 35, and 42 post-electroporation. For (C), data represent mean ± SEM of 3 independent replicates. ∗∗∗*p* < 0.001, ∗∗∗∗*p* < 0.0001. *p* values were determined using one-way ANOVA analysis. (D) Cell viability analysis of all the groups of mock, *CISH*^KO^, and CD19CAR NK cells over the course of 42 days *in vitro* culture and expansion post-electroporation. Data represent mean ± SEM of 2 independent replicates. (E) The expansion potential of mock, *CISH*^KO^, and CD19CAR NK cells over the course of 42 days *in vitro* culture and expansion post-electroporation. Data represent mean ± SEM of 2 independent replicates. (F and G) *In vitro* cytotoxicity of the engineered CD19CAR NK cells against CD19-positive NALM6 target cells co-cultured at various E:T ratios for 4 h (F: day 21 post-electroporation; G: day 35 post-electroporation). Data represent mean ± SEM of 3 independent replicates. ∗*p* < 0.05, ∗∗*p* < 0.01, ∗∗∗*p* < 0.001, ∗∗∗∗*p* < 0.0001. *p* values were determined using one-way ANOVA analysis.

To knock in a functional CAR into the *CISH* locus, we then synthesized a CD19CAR cssDNA donor template flanked by ∼350 nt 5′ and ∼350 nt 3′ homology arms targeting the *CISH* locus (Figure 2A). The cssDNA donor template was then co-delivered with the RNP complex into NK cells using the Lonza 4D nucleofector electroporation system. As shown in Figures 2B and 2C, 7 days post-electroporation, a ∼10% KI efficiency (CD19CAR expression) was achieved using a 2 μg/reaction dosage of the cssDNA donor template. Notably, the CAR expression level continued to increase throughout *ex vivo* NK cell expansion, rising from an average of 9.7%–41.5%. This CAR enrichment phenotype, driven by *CISH* locus targeted engineering, was not observed at other loci such as *RAB11A*. Specifically, in an attempt to integrate CD19CAR in NK cells from two independent donors into the *RAB11A* locus, we observed a stable CD19CAR-expressing population at both day 7 and day 21 post-electroporation, with donor #1 NK cells averaging ∼20% and donor #2 NK cells averaging ∼10% CAR expression using 2 μg of cssDNA donor template per reaction (Figures S2W–S2Z).

Cell viability measurements across all groups indicated that cssDNA-mediated KI at the applied dosage (2 μg/reaction) was biocompatible and did not induce significant cytotoxicity (Figure 2D). The low viability observed on day 1 was attributed to transient physical impairment caused by electroporation. However, all NK cell groups recovered to ≥90% viability after 6 days in culture. Moreover, NK cells with *CISH* knockout (both RNP-only and CD19CAR-KI) maintained high viability for an extended period, remaining at ≥90% viability up to 35 days post-electroporation. In contrast, mock NK cells exhibited a much shorter duration of high viability, followed by a sharp decline, averaging 55% viability on day 35. Additionally, the cell proliferation curve demonstrated that *CISH* knockout substantially improved NK cell persistence and expansion over an extended culture period (Figure 2E).

Regarding anti-tumor activity, we assessed the cytolytic function of NK cells against NALM6 target cells at two time points: 21 days and 35 days post-electroporation. As shown in Figures 2F and 2G, engineered CD19CAR-NK cells exhibited the highest cytolytic activity among all groups. Furthermore, at the later time point (day 35 post-electroporation), the cytolytic activity of all NK cell groups declined to varying degrees compared to day 21. Notably, mock NK cells showed the most significant reduction in cytotoxicity (from an average of 38%–3% at an effector-to-target (E:T) ratio of 1:1 and from 63% to 19% at an E:T ratio of 2:1), indicating exhaustion after prolonged *ex vivo* culture. In contrast, *CISH* ^KO^ (RNP-only) NK cells and CD19CAR-NK cells maintained substantial cytolytic activity, with CD19CAR-NK cells demonstrating the strongest tumor-killing effect even at day 35 post-electroporation.

To further evaluate the robustness of CLICK-mediated CD19CAR-NK engineering across different donor NK cells, we performed KI experiments on NK cells from donor #2. The initial KI efficiency of CD19CAR at the CISH locus was 13%, with CAR expression gradually increasing and plateauing at approximately 64% by day 35 post-electroporation (Figures S4A and S4B). Consistent with donor #1, *CISH* knockout NK cell groups (RNP-only and CD19CAR-NK) maintained high viability and exhibited enhanced persistence and proliferation during extended culture period (Figures S4C and S4D). Moreover, CD19CAR-NK cells from donor #2 displayed robust cytotoxicity against NALM6 target cells at all assessed time points—early (days 7 and 21) and late (day 35)—with activity peaking at day 21 and showing a slight decline thereafter despite sustained high CAR expression (Figures S4E–S4G). These findings suggest that while cellular exhaustion over time is inevitable, the combined effects of CAR expression and *CISH* knockout mitigate functional decline and sustain potent anti-tumor activity.

### Engineering and characterization of CLICK CD19CAR/sIL-15-NK

**Figure 3:**
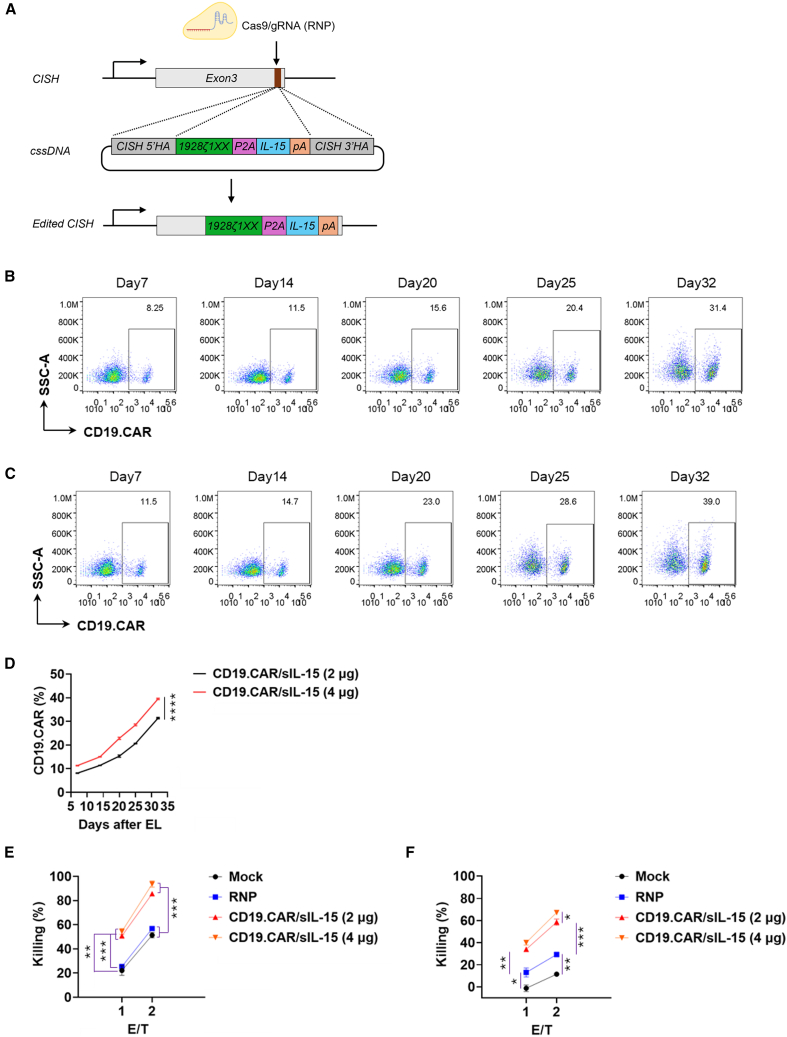
Functional CD19CAR/sIL-15 CAR-NK engineering by CLICK (A) Schematic diagram of cssDNA donor template used for the genome insertion of CD19CAR/sIL15 (1928ζ1XX/P2A/sIL-15) gene at *CISH* locus. (B–D) Representative flow cytometry profiles (B and C) and quantifications (D) of CD19CAR/sIL15 knock-in efficiency at *CISH* locus (CD19CAR %) on day 7, 14, 21, 28, 35, and 42 post-electroporation. Here, cssDNA donor template was used for NK cell engineering at 2 and 4 μg/reaction for (B) and (C), respectively. For (D), data represent mean ± SEM of 3 independent replicates. ∗∗∗∗*p* < 0.0001. *p* values were determined using the two-tailed Student’s *t* test analysis. (E and F) *In vitro* cytotoxicity function of the engineered CD19CAR/sIL15 NK cells against CD19-positive NALM6 target cells co-cultured at various E:T ratios for 4 h (F: day 17 post-electroporation; G: day 32 post-electroporation). Data represent mean ± SEM of 3 independent replicates. ∗*p* < 0.05, ∗∗*p* < 0.01, ∗∗∗*p* < 0.001. *p* values were determined using one-way ANOVA analysis.

Engineered NK cells expressing CD19CAR and soluble secreted interleukin-15 (CD19CAR/sIL-15) by virus-based genome editing have been evaluated in preclinical models and clinical trials for the treatment of CD19^+^ B cell malignancies, demonstrating favorable safety and efficacy profiles.^9^^,^^44^^,^^45^ We next sought to determine whether the CLICK platform, utilizing a cssDNA HDR donor template, could similarly generate clinically relevant CD19CAR/sIL-15 NK cells with an expanded payload size. To this end, we synthesized a cssDNA donor template containing a CD19CAR and soluble IL-15 co-expression cassette (CD19CAR/sIL-15), separated by a 2A peptide sequence. The insertion payload was flanked by ∼350 nt 5′ and ∼350 nt 3′ homology arms targeting the *CISH* locus, with a total cssDNA payload size of approximately 3.5 kb. The cssDNA donor template was then co-delivered with the RNP complex into NK cells using the Lonza 4D nucleofector electroporation system at two dosages: 2 μg/reaction and 4 μg/reaction (Figure 3A).

Results indicated that cssDNA HDR donor template-mediated engineering produced CD19CAR expression in a dose-dependent manner, with higher expression observed at 4 μg/reaction compared to 2 μg/reaction. Moreover, the percentage of CD19CAR^+^ NK cells continued to increase as NK cells expanded and proliferated, reaching ∼31% by day 32 for the 2 μg cssDNA condition and ∼40% for the 4 μg condition, compared to ∼8% and ∼11% expression on day 7, respectively (Figures 3B–3D). Regarding cytolytic activity, engineered CD19CAR/sIL-15 NK cells exhibited significantly enhanced *ex vivo* cytotoxicity against NALM6 target cells compared to both mock and RNP-only NK cells at two evaluated time points—early (day 17) and late (day 32)—post-electroporation. The cytolytic effect was relatively higher in NK cells engineered with the 4 μg/reaction dosage than in those engineered with 2 μg/reaction at both time points (Figures 3E and 3F). Consistent with the findings from CD19CAR-only NK cells, cytolytic activity across all NK cell groups declined to varying degrees at the later time point (day 32) compared with earlier measurements. Specifically, ock group cells exhibited the most pronounced reduction in cytotoxicity, whereas *CISH* knockout (RNP-only) NK cells and CD19CAR/sIL-15 NK cells retained substantial tumor-killing capacity.

In an independent NK cell donor (donor #2), we similarly observed an increasing CAR^+^ population over the course of NK cell expansion. However, baseline engineering efficiency was comparatively lower at both the 2 μg and 4 μg cssDNA template dosages than in donor #1 (Figures S5A–S5C). Consistent with the first donor, engineered CD19CAR/sIL-15 NK cells demonstrated the highest *ex vivo* cytotoxicity against NALM6 target cells compared with mock and RNP-only groups, with the 4 μg cssDNA condition showing superior activity to the 2 μg condition at the evaluated time point (Figure S5D).

These data demonstrate that the CLICK non-viral engineering platform enables the integration of multi-kilobase polycistronic functional transgene cassettes, efficiently generating clinically relevant CD19CAR/sIL-15 NK cells with a robust KI efficiency specifically at the *CISH* locus and potent *ex vivo* anti-tumor activity. Furthermore, CAR^+^ population enrichment was consistent across different payload sequences and lengths.

### Feeder-free culture system is compatible for CLICK non-viral CAR-NK engineering

**Figure 4:**
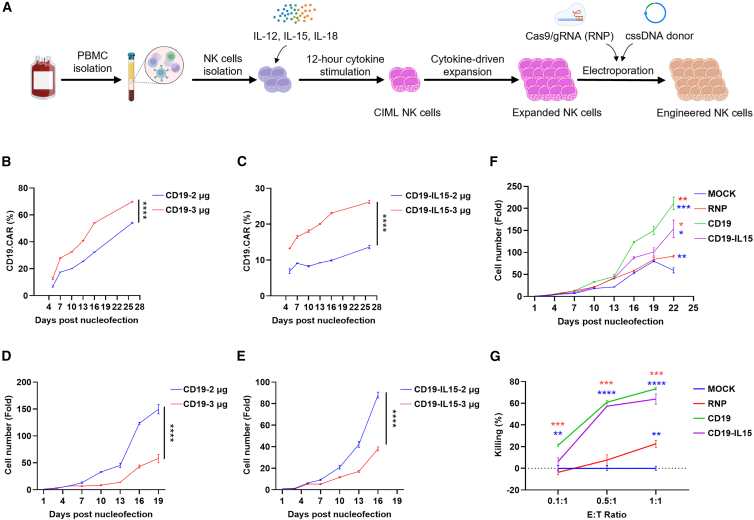
CLICK CD19CAR-NK engineering at *CISH* locus with feeder-free system (A) Schematic diagram of the feeder-free *ex vivo* expansion and CRISPR-Cas9-mediated gene editing strategy for NK cell engineering at the *CISH* locus. (B) Positive rate of CD19CAR knock-in over time for NK cell engineered with 2 μg and 3 μg cssDNA donor template doses. (C) Positive rate of CD19CAR/sIL-15 knock-in over time for NK cell engineered with 2 μg and 3 μg cssDNA donor template doses. (D) Proliferation fold expansion of CD19CAR NK cells over time with 2 μg and 3 μg cssDNA donor template doses. (E) Proliferation fold expansion of CD19CAR/sIL-15 NK cells over time with 2 μg and 3 μg cssDNA donor template doses. (F) Proliferation fold of mock, RNP (*CISH*^KO^), CD19CAR (2 μg group), and CD19CAR/sIL-15 (2 μg group) NK cells over time. (G) Killing rate of mock, RNP (*CISH*^KO^), CD19CAR, and CD19CAR/sIL-15 NK cells by luciferase assay. RNP (*CISH*^KO^) NK cells were added to CD19CAR NK cells to make the positive rate of CAR expression same with CD19-CAR/IL-15. The results of luciferase activity were normalized to mock NK cells to exhibit specific killing of other groups. Data in (B)–(G) represent mean ± SEM of 3 independent replicates. ∗*p* < 0.05, ∗∗*p* < 0.01, ∗∗∗*p* < 0.001, ∗∗∗∗*p* < 0.0001. *p* values in (B)–(E) were determined using the two-tailed Student’s *t* test analysis. *p* values in (F) and (G) were determined using one-way ANOVA analysis.

Although NK cells expanded using feeder cell systems achieve high purity and robust expansion, their clinical application is associated with safety concerns.^46^ To address this issue, a feeder-free culture system was employed to evaluate the efficiency of NK cell engineering using cssDNA in NK cells from an additional donor (donor #3) (Figure 4A). A total of 2 μg or 3 μg of cssDNA encoding CD19CAR or CD19CAR/IL-15 was co-electroporated with the RNP complex into freshly isolated PB-NK cells. Consistent with previous findings, the CLICK platform successfully facilitated NK cell engineering, generating CAR-NK cells with *CISH* knockout. Flow cytometry analysis on day 7 post-electroporation revealed CAR^+^ NK cell frequencies of 28% and 16% for CD19CAR and CD19CAR/IL-15 with 3 μg of cssDNA donor template, respectively. Similar to observations in the feeder cell system, CAR^+^ populations increased over time, with significantly higher KI efficiency achieved using 3 μg of cssDNA compared to 2 μg (Figures 4B and 4C). However, NK cells in the 2 μg cssDNA group exhibited superior proliferation potential compared to those in the 3 μg group (Figures 4D and 4E), suggesting that higher cssDNA doses may partially impair NK cell proliferation in NK donor #3.

Compared to mock NK cells, RNP-treated (*CISH* ^KO^) NK cells, CD19CAR NK cells, and CD19CAR/IL-15 NK cells all demonstrated enhanced proliferation (Figure 4F), highlighting the beneficial effects of *CISH* knockout and CAR expression. To further assess the cytotoxic potential of engineered CAR-NK cells, different NK cell groups were co-cultured with CD19^+^ NALM6-luc-GFP cells at varying E:T ratios for 5 h. Luciferase-based cytotoxicity assays confirmed that CD19CAR NK cells and CD19CAR/IL-15 NK cells exhibited significantly higher cytotoxic activity compared to mock or *CISH* ^KO^ NK cells. Notably, *CISH* ^KO^ alone also conferred enhanced cytotoxicity relative to mock NK cells at high E:T ratios, consistent with those found with feeder culture system (Figure 4G). In summary, these findings demonstrate that the CLICK non-viral gene engineering platform is well suited for integration into a feeder-free NK cell culture workflow, further supporting its versatility and potential clinical applicability.

### CLICK CD19CAR-NK enhances the antitumor activity with improved leukemia control

**Figure 5:**
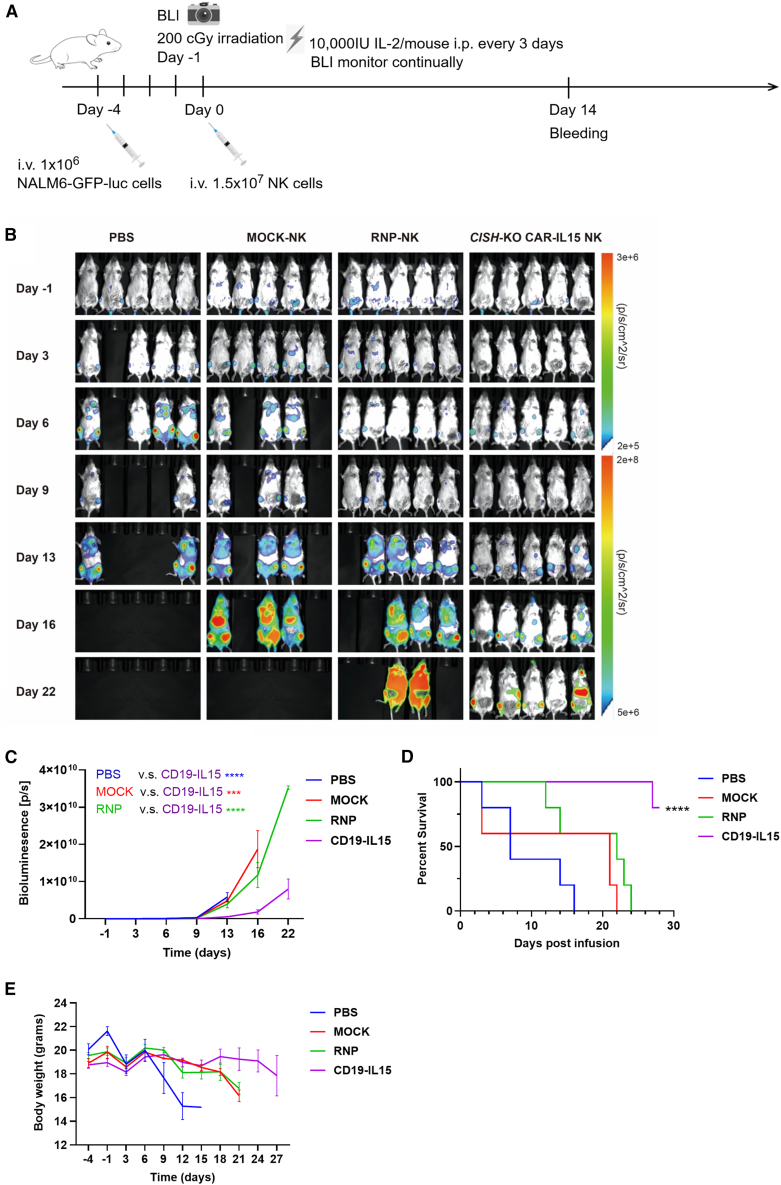
*In vivo* function of CD19CAR-NK engineering at *CISH* locus by CLICK (A) Schematic diagram of the experimental process for CAR-NK *in vivo* functional analysis. (B) Tumor burden was determined by BLI over time. (C) Quantification of tumor burden from total flux of (B). (D) Kaplan-Meier survival curves of mice. (E) The body weights of mice over time in the 4 different groups. Data in (C) and (E) represent means ± SEM. ∗∗∗*p* < 0.001 and ∗∗∗∗*p* < 0.0001. *p* values in (C) were determined using one-way ANOVA analysis. *p* values in (D) were determined using log rank test.

Given the promising *ex vivo* results, we further evaluated the anti-tumor efficacy of the CLICK engineered CD19CAR/sIL-15 NK cells in a NALM6 lymphoblastic leukemia model using NOD/ShiLtJGpt-*Prkdc*^em26Cd52^*Il2rg*^em26Cd22^/Gpt (NCG) mice. Mice were intravenously injected with GFP/luciferase-expressing NALM6 cells for tumor inoculation (1 × 10^6^ cells/mouse) (Figure 5A). Three days later, successful engraftment was confirmed by bioluminescence imaging (BLI), followed by 200 cGy radiation conditioning. The next day, mice received a single intravenous injection of mock NK cells, RNP-treated (*CISH* ^KO^) NK cells, or *CISH* ^KO^/CD19CAR/sIL-15 NK cells (1.5 × 10^7^ cells/mouse), while the control group received PBS. To further support NK cell survival and expansion *in vivo*, IL-2 was administered intraperitoneally every 3 days. Tumor burden was monitored every 3 or 4 days via BLI.

As shown in Figures 5B–5E, mice treated with *CISH* ^KO^ NK cells exhibited a moderate reduction in leukemia burden compared to those treated with mock NK cells or PBS. Notably, mice receiving CD19CAR/sIL-15 NK cells demonstrated a significant reduction in leukemia burden, leading to prolonged survival compared to those treated with *CISH* ^KO^ NK cells alone or mock NK cells. These results are consistent with our *ex vivo* findings, further supporting that CLICK engineered CD19CAR/sIL-15 NK cells exhibit enhanced anti-NALM6 cytotoxicity *in vivo*.

**Figure 6:**
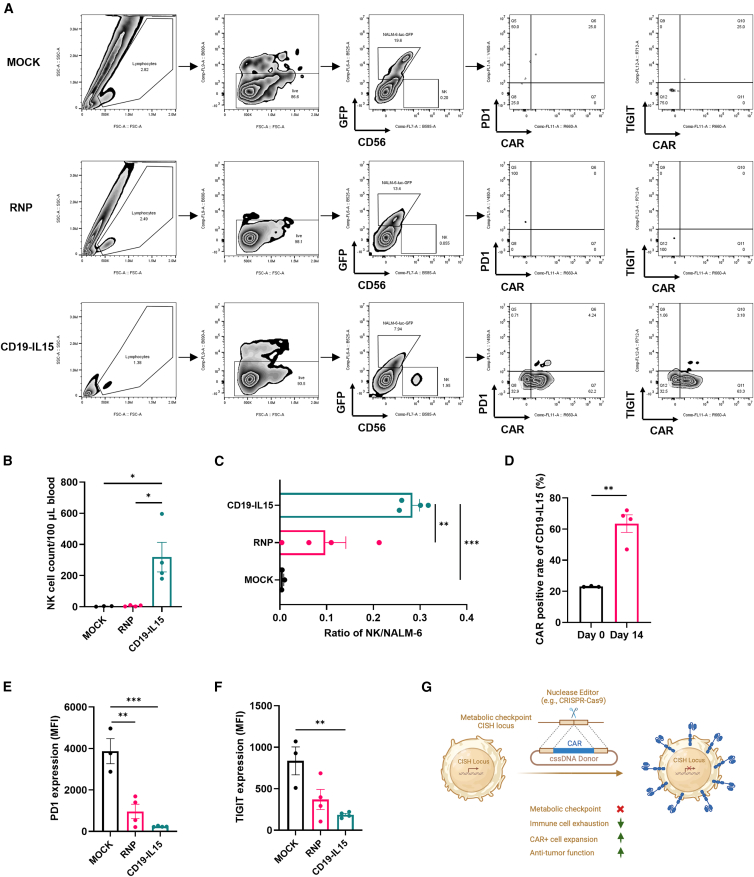
Enhanced non-viral CD19CAR-NK persistence at *CISH* locus *in vivo* (A) Representative flow cytometry plots showing the gating strategy and expression profiles. (B) Comparison of total numbers of human NK (hNK) cells in peripheral blood between mice injected with different hNK cells. (C) The ratio of hNK cells/NALM-6 cells in peripheral blood from the treated mice. (D) Comparison of the percentage of CAR-positive NK cells in total hNK cells in peripheral blood of mice between day 0 and day 14 post-engraftment. (E and F) Mean fluorescence intensity (MFI) of (E) PD1 and (F) TIGIT. (G) Schematic diagram of the CLICK engineering platform for NK cells and the resulting cellular and functional phenotypes. NK cells are engineered using non-viral cssDNA to integrate a specific CAR transgene at the metabolic CISH locus, thereby simultaneously ablating CISH protein expression and knocking in a functional chimeric antigen receptor. The engineered NK cells exhibit improved expansion, reduced exhaustion *ex vivo* and *in vivo*, and enhanced antitumor activity. Data in (B)–(F) represent means ± SEM of three or four independent replicates. ∗*p* < 0.05, ∗∗*p* < 0.01, ∗∗∗*p* < 0.001. *p* values in (B), (C), (E), and (F) were determined using one-way ANOVA analysis. *p* values in (D) were determined using the two-tailed Student’s *t* test analysis.

Next, we investigated the persistence and viability of CD19CAR/IL-15 NK cells *in vivo*. Peripheral blood was collected from the tail vein of treated mice on day 14 post-NK cell engraftment and analyzed by flow cytometry for human NK cell abundance. Significantly higher NK cell counts and NK/leukemia ratios were detected in 100 μL of blood from mice treated with CD19CAR/sIL-15 NK cells compared to those treated with other NK cell groups (Figures 6A–6C). Interestingly, the proportion of CAR^+^ NK cells was notably increased to >60% compared to day 0 at around 22%, suggesting that CAR-NK cells were enriched when the CAR transgene was integrated into the *CISH* locus (Figure 6D). Notably, 4.36% of CAR^+^ NK cells remained detectable 22 days post-engraftment (Figure S6).

Additionally, we analyzed the expression of PD-1 and TIGIT, two immune checkpoint markers associated with NK cell exhaustion, in NK cells recovered from mice receiving different treatments. As shown in Figures 6E and 6F, CD19CAR/sIL-15 NK cells exhibited lower PD-1 and TIGIT expression levels than NK cells with RNP (*CISH* ^KO^) or mock NK cells. Overall, these findings provide strong evidence for the enhanced anti-leukemia efficacy and prolonged *in vivo* persistence of CLICK-engineered CD19CAR/sIL-15 NK cells, highlighting the therapeutic potential of this approach.

## Discussion

### cssDNA non-viral engineering for precise NK cell modification

NK cells are increasingly recognized for their therapeutic potential in immunotherapy due to their intrinsic cytotoxicity without prior antigen sensitization and their favorable safety profile with minimal risk of cytokine release syndrome, neurotoxicity, and GvHD. However, efficient genetic modification of NK cells remains challenging due to their resistance to exogenous DNA uptake and limited persistence at tumor sites. Traditional viral-based gene delivery systems, such as lentiviral or retroviral vectors, face several limitations, including inconsistent transduction efficiency, restricted cargo capacity, manufacturing complexity, and safety concerns related to random insertional mutagenesis. Therefore, developing precise and safe genetic tools is essential to unlock the full therapeutic potential of NK cells.

In this study, we introduce CLICK , a non-viral, targeted genome integration platform leveraging phagemid-derived mini-cssDNA as a donor template for HDR. This approach significantly advances the field of NK cell engineering by addressing the inherent challenges associated with viral and other non-viral delivery systems. Unlike conventional double-stranded DNA templates that are cytotoxic and inefficient in NK cells, the cssDNA-based method offers high KI efficiency with minimal cytotoxicity. Furthermore, the circular nature of cssDNA enhances its stability and integration precision, thereby reducing the risk of random mutagenesis. These advantages underscore the superiority of the cssDNA platform in achieving precise and safe genetic modifications in primary NK cells.

### Desirable phenotypes of NK cells with two-in-one CLICK strategy

One of the most innovative aspects of the CLICK platform is its two-in-one strategy, which simultaneously disrupts the *CISH* locus while integrating functional CAR constructs. *CISH* encodes the CIS protein, a key metabolic checkpoint that negatively regulates NK cell function. By targeting the *CISH* locus with an optimal targeting sgRNA, we not only effectively eliminate this inhibitory checkpoint but also leverage the highly accessible chromatin environment of this locus for efficient site specific non-viral CAR integration. This dual action enhances NK cell persistence, proliferation, and cytotoxicity, as demonstrated by the increased CAR-NK expansion, enriched CAR^+^ population and superior anti-tumor activity observed during *ex vivo* and *in vivo* studies (Figure 6G). The enrichment of CAR-positive NK cells during *ex vivo* expansion further highlights the advantage of this two-in-one engineering strategy, potentially reducing the need for extensive cell sorting and purification. This strategic approach, combining functional gene knockout with targeted CAR integration in a single step, provides a streamlined manufacturing process option for off-the-shelf allogenic immune cell therapy.

### Modular feature of CLICK mediated functional immune cell engineering

The versatility of the CLICK platform extends beyond CD19 CAR engineering. Its flexible and modular design enables the integration of diverse multi-kilobase therapeutic payloads, such as soluble cytokines (e.g., sIL-15) or bispecific targeting moieties, thereby enhancing NK cell functionality and persistence. As a proof of concept, we here successfully generated CD19 CAR/sIL-15-expressing NK cells, which demonstrated potent antitumor activity and prolonged survival in a leukemia mouse model.

The potential applications of this integrated two-in-one strategy are broad, spanning both hematologic malignancies and solid tumors. Importantly, the CLICK platform can be readily adapted to disrupt additional inhibitory checkpoints or to introduce supplementary functional payloads, enabling the rational design of next-generation NK cell therapies tailored to specific cancer types or immunosuppressive tumor microenvironments. Notably, several additional gene targets—including *MED12*, *CCNC*, and *ARIH2*—have recently been identified as critical regulatory checkpoints in primary human NK cells that govern resistance to immunosuppressive signals and ablation these genes significantly improved NK cell antitumor activity against multiple treatment-refractory human cancers *in vitro* and *in vivo*.^47^ The modular nature of cssDNA donor templates facilitates customization and scalable implementation, enabling systematic evaluation of combinatorial gene-editing strategies in NK cells.

In conclusion, the CLICK platform provides a robust and flexible solution for precise, non-viral genetic engineering of NK cells, addressing key challenges associated with traditional viral vectors. Its innovative two-in-one strategy effectively combines checkpoint inhibition with CAR integration, resulting in enhanced NK cell persistence, proliferation, and cytotoxicity. By demonstrating the feasibility and efficacy of this approach, our study lays the foundation for the development of next-generation NK cell therapies with broad-spectrum applications in cancer and other diseases. Future advancements in cssDNA design, delivery optimization, and combinatorial gene editing will further accelerate the translation of CLICK platform technology.

## Materials and methods

### cssDNA donor production

Detailed method for cssDNA production was described previously.^25^^,^^48^ Specifically, donor template sequences (transgene sequence flanked with 5′ and 3′ homology arms at 300–500 nt in length) are constructed as dsDNA phagemid vector. Host XL1-Blue *E. coli* strain was co-transformed with the M13 helper plasmid and phagemid containing double-stranded donor template and selected on agar plates with kanamycin (50 μg/mL) and carbenicillin (100 μg/mL). A single colony was selected and grown for ∼24 h (37°C, 225 rpm) in 250 mL 2×YT media (1.6% tryptone, 1% yeast extract, and 0.25% NaCl) to reach OD_600_ between 2.5 and 3.0. The bacteria were pelleted by centrifugation, and the phage particles in the supernatant were precipitated with PEG-8000. The precipitated phage particles were then pelleted by centrifugation, washed, and lysed in 20 mM MOPS, 1 M Guanidine-HCl, and 2% Triton X-100. The cssDNA released from the phage were then extracted with NucleoBond Xtra Midi EF kit (Macherey-Nagel) following the manufacturer’s instructions. The concentration of cssDNA was determined by Nanodrop specific for ssDNA. The cssDNA product sequences were verified by Sanger sequencing or nanopore sequencing.

### Cell culture

K562 (ATCC, CCL-243), NALM6 (ATCC, CRL-3273), and Epstein-Barr virus-transformed lymphoblastoid cell line (EBV-LCL) (ATCC, CRL-5962) cells were maintained in RPMI-1640 media (Gibco) with 10% FBS (Gibco) and 100 U/mL penicillin-streptomycin (Gibco). Primary human NK cells were isolated from fresh human peripheral blood Leukopak by either negative selection using the EasySep Human NK cell Isolation Kit (STEMCELL Technologies), or positive selection using NK Cell Isolation Kit, human (Miltenyi Biotec). Freshly isolated NK cells can be directly expanded or frozen in CryoStor CS10 Cell Freezing Medium (10^7^ cells/mL per cryogenic tube) and stored in a liquid N_2_ tank to be thawed and expanded later. NK cells were expanded through co-culture with EBV-LCL feeder cells at a 1:10 (NK cell: feeder cell) ratio in NK MACS medium (Miltenyi Biotec) supplemented with 5% heat inactivated human AB serum (Valley Biomedical), 500 IU/mL rhIL-2 (PeproTech), and 100 ng/mL rhIL-21 (PeproTech). Herein, the rhIL-21 was added only once at the beginning of the co-culture. Prior to co-culture, feeder cells were treated with 10 μg/mL of mitomycin C (Thermo Fisher Scientific) for 3 h to inhibit proliferation, followed by washing with 1× PBS (Gibco). GFP and firefly luciferase dual-reporter-expressing NALM6 cells (designated as NALM6-GFP-luc) were engineered internally with plasmid transfection and clonal isolation. NALM6-GFP-luc cells are cultured in RPMI-1640 media supplemented with 10% FBS and 100 U/mL penicillin-streptomycin. All cells were incubated at 37°C in a humidified 5% CO_2_ environment. Cell counts as well as the cell viability were determined using a Via2-Cassette in NucleoCounter NC-202 (ChemoMetec) on specified days.

### Electroporation

Electroporation was performed using the Amaxa 96-well Shuttle with the 4D-Nucleofector X Unit (Lonza). The P3 Primary Cell 4D-Nucleofector Kit (Lonza) was used for transfection of NK cells. Here, ∼500,000 NK cells were used per reaction with the program code DN-100. Cas9 RNP complex was prepared using 25 picomole of sNLS-SpCas9-sNLS Nuclease (Aldevron) along with 50 picomol of sgRNA (Integrated DNA Technologies) per reaction. Cas9-sgRNA RNP) complexes were assembled in P3 buffer for 20 min at room temperature. For the gene KO (e.g., *CISH*), NK cells were resuspended in P3 buffer and mixed with RNP complexes prior to electroporation. After electroporation, the cells were transferred into NK MACS complete medium (with 5% heat inactivated human AB serum and 500 IU/mL rhIL-2) for continuous culture. For specific constructs KI (e.g., CD19CAR and CD19CAR/sIL-15), the indicated amount of HDR donor templates carrying gene of interest (GOI) were mixed with the RNP complexes and co-electroporated into the NK cells. Following electroporation, the cells were transferred into NK MACS complete medium supplemented with 1 μM HDR enhancer M-3814 (MedKoo Biosciences) and incubated for 24 h. Subsequently, the M-3814 was removed, and the cells were cultured under NK MACS complete medium.

### Gene-editing efficiency analysis

RNP-mediated gene cutting efficiency (Indel %) was measured by NGS amplicon sequencing. Briefly, the edited cells were collected, and the genomic DNA was extracted using PureLink Genomic DNA Kits (Invitrogen). Target regions were amplified by PCR using KAPA HiFi HotStart PCR kit (Roche Diagnostics) with the primer sets listed in Table S1. The PCR products were purified by using AMPure XP Beads (Beckman Coulter) and eluted in molecular-grade water for NGS amplicon sequencing in NGS division at Quintara Biosciences. The indel percentage was analyzed through CRISPResso2 online software. Target gene-mediated protein expression (CISH protein) was tested by western blot. Briefly, NK cells were collected and lysed in RIPA lysis buffer system (Santa Cruz Biotechnology). Protein concentrations were determined by Pierce BCA Protein Assay Kits (Thermo Fisher Scientific). The following primary antibodies were used: CISH antibody (Clone D4D9) and GAPDH antibody (Clone 14C10), both obtained from Cell Signaling Technology. Expression bands were imaged and captured by iBright CL1000 Imaging System (Invitrogen). KI efficiency, as indicated by CD19CAR expression, was measured using flow cytometry. For CAR staining, an Alexa Fluor 647 AffiniPure F(ab′)_2_ Fragment Goat Anti-Mouse IgG, F(ab′)_2_ fragment specific antibody was used (Jackson ImmunoResearch). SYTOX Blue Dead Cell Stain (Invitrogen) was used to exclude the dead cells.

### *Ex vivo* killing assay

To test for killing potency of NK cells, a cytotoxicity assay was carried out. Briefly, NK cells were co-cultured with target cells (K562 or NALM6) at various E:T ratios for ∼4 h. The killing ability of the effector cells against target cells was measured via a CCK-8 assay according to the manufacturer’s instructions. Absorbance was measured at 450 nm using a microplate reader (SpectraMax, M2). The cytotoxicity was calculated as follows: Cytotoxicity (%) = 100% – [(OD_E+T_ – OD_B_) – (OD_E_ – OD_B_)]/(OD_T_ – OD_B_) × 100% (OD, absorbance; E, effector; T, target; and B, blank).

### Feeder-free primary PB-NK cell culture

Primary NK cells were isolated from healthy donor PBMCs using STEMCELL EasySep Human NK Cell Isolation Kit (Cat# 17955). Freshly isolated NK cells were activated in a medium containing three cytokines (IL-12, IL-15, and IL-18) for 12 h, leading to cytokine-induced memory-like (CIML) NK cell differentiation.^30^ Following activation, NK cells were cultured and expanded in OptiVitro NK cell medium (ExCell Bio, Cat# NE000-N022). Seven days later, NK cells were ready for nucleofection-mediated genetic engineering.

### Flow cytometry

Single-cell suspensions were stained with the appropriate antibody in PBS containing 2% FBS on ice for 30 min. Anti-CD56, anti-PD1, anti-TIGIT, and anti-streptavidin were purchased from BioLegend. Biotinylated recombinant human CD19 protein was purchased from Sino Biological. 7-AAD was purchased from Yeasen. The flow cytometry data were collected on CytoFLEX (Beckman) and analyzed by FlowJo v.10.8.1.

### Luciferase assay

To assess the cytolytic function of CAR-NK cells against target cells, CAR-NK cells were co-incubated with luciferase expressing NALM6 cells at the indicated E:T ratios. After 5 h of co-culture, relative luciferase activities were measured using the Promega Luciferase Assay System (Cat# E1500) according to the manufacturer’s instructions. Specific lysis of each group was calculated using the following formula: lysis (%) = (1−luciferase units of RNP (*CISH* ^KO^) NK cell or CAR-NK cell group/luciferase units of mock NK cell group) × 100.

### Animal study

NCG mice were purchased from GemPharmatech Co., Ltd. and housed in standard specific pathogen-free rearing center. The animal experiments were approved by the Institutional Animal Care and Use Committee of Nanfang Hospital (Guangzhou, China) and complied to the rules required by the Guide for the Care and Use of Laboratory. NCG mice were injected with GFP and luciferase co-expressing NALM-6 cells (1 × 10^6^) intravenously (day −4) and imaged for engraftment 3 days later and received 200 cGy radiation (day −1). On day 1, mice were then injected intravenously with 1.5 × 10^7^ mock NK cells, RNP (*CISH* ^KO^) NK cells, CD19CAR/sIL15 NK cells, or PBS. From day 1, mice were intraperitoneally administered 10,000 IU IL-2 (Gibco, Cat# 200–02) every 3 days until day 14 post NK injection. Tumor burden was continually monitored by BLI (Ami HTX Imaging System). On day 14 post-infusion of NK cells, peripheral blood samples were collected for flow cytometry analysis. Health of mice was monitored by body weight measurement and assessment of signs of pain and distress. Mice were euthanized when they met pre-defined criteria defined in the animal license.

### Statistical analysis

Data are presented as mean ± SEM. Statistical analysis was performed using Excel software (Office 365, Microsoft) and Prism 10 (GraphPad). Comparison between two normally distributed test groups was performed using the two-tailed Student’s *t* test. For analysis of three or more groups, comparison was performed using a one-way ANOVA analysis. *p* < 0.05 was considered statistically significant.

## Data and code availability

The data presented in this study are available from the corresponding authors upon reasonable request.

## Acknowledgments

We thank Quintara Bioscience Inc. for providing cloning and sequencing services and Danna Lee from Full Circles Therapeutics Inc. for technical assistance with DNA vector purification. This work was supported by the Hospital-Industry Collaboration Project (GRGZ2022330 and GRGZ20230520), the 10.13039/501100001809National Natural Science Foundation of China (82373302), the Science and Technology Projects of Guangzhou (2024A04J6613 and 2023A04J2362), and the Science and Technology Plan Project of Guangdong (2024A0505040015).

## Author contributions

J.W. and H.W. conceived the study; J.W., K.X., Y.S., G.X., and H.W. designed the experiments and interpreted the data; J.W., Y.S., J.S., F.W., and X.D. performed the experiments; G.X. and H.W. supervised the project; J.W., Y.S., G.X., and H.W. wrote the manuscript with input from all authors.

## Declaration of interests

K.X., J.S., J.W., R.S., X.H., and H.W. are either current or former employees of Full Circles Therapeutics, INC. Patents related to this study have been filed by Full Circles Therapeutics, INC.

## Supplemental information

Document S1. Figures S1–S6 and Table S1

Document S2. Article plus supplemental information
