# Supplementary material for: Non-viral targeted integration at the CISH locus enables CAR-NK cell engineering with enhanced anti-tumor activity
Source: Mol Ther Oncol. 2026 Apr 17;34(2):201206. doi: 10.1016/j.omton.2026.201206 (PMC13137893; doi:10.1016/j.omton.2026.201206)
Supplement: Document S1. Figures S1–S6 and Table S1 [file mmc1.pdf]

OMTON, Volume *34*

Supplemental information

Non-viral targeted integration at the CISH locus enables CAR-NK cell engineering with enhanced anti-tumor activity

Jiao Wang, Yao Sun, Jakob Starzyk, Fei Wang, Xin Dong, Richard Shan, Xuemei He, Keqiang Xie, Guozhu Xie, and Hao Wu

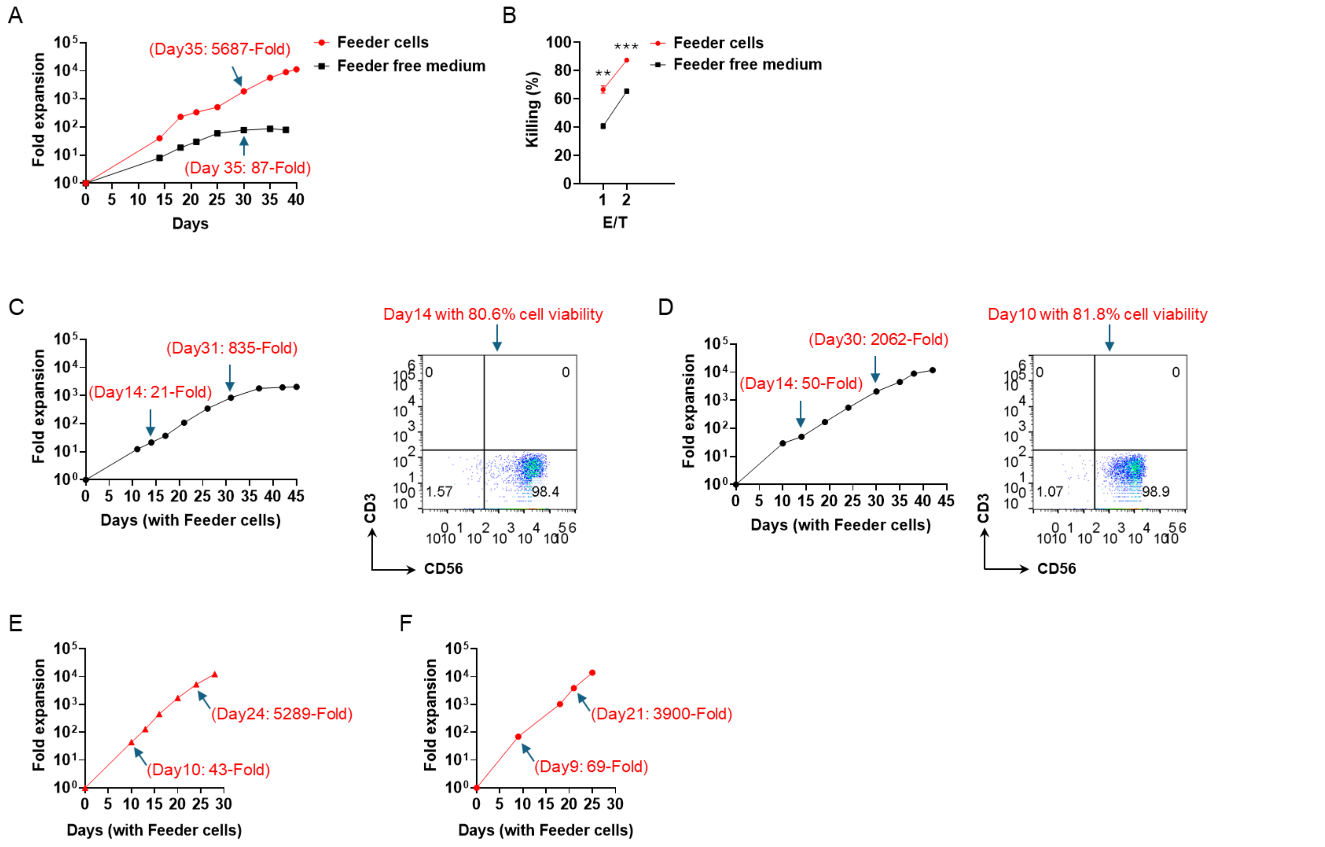

Figure S1: NK cells *in vitro* expansion with EBV-LCL feeder cells

A&B. EBV-LCL feeder cells mediated NK cells expansion exhibited superior performance, including robust and sustained proliferation (left panel, A) and enhanced *in vitro* cytotoxicity against K562 cells (right panel, B). The cryopreserved NK cells used here were derived from one representative donor. The NK cells used for the *in vitro cytotoxicity* were harvested on Day 28 of expansion. For B, data represent mean ± SEM of 3 independent replicates. **P< 0.01, **P<0.001. P values were determined using the two-tailed Student's *t*-test analysis.

C&D. The established two-phase expansion method for cryopreserved NK cells—Phase 1: thawing, resting, and recovery in NK MACS medium for around 1 week; Phase 2: co-culture expansion with EBV-LCL feeder cells—demonstrated a high and sustained proliferation rate. (C) The expansion profile of the cryopreserved NK cells co-cultured with feeder cells on Day 0 after thaw. Herein, on Day 14, the feeder cells in the co-culture system got cleared showing >80% cell viability and >95% purity ($CD56^+CD3^-$). (D) The expansion profile of the cryopreserved NK cells cultured with feeder cells on Day 7 post-thaw, following 1 week of rest and recovery in culture medium. Herein, on Day 10, the feeder cells in the co-culture system got cleared showing >80% cell viability and >95% purity ($CD56^+CD3^-$). The cryopreserved NK cells used here were derived from one representative donor.

E&F. The defined two-phase expansion method enabled (E) cryopreserved NK cells to achieve a proliferation rate comparable to that of (F) freshly isolated NK cells co-cultured with EBV-LCL feeder cells from Day 0. The NK cells used here were derived from one representative donor.

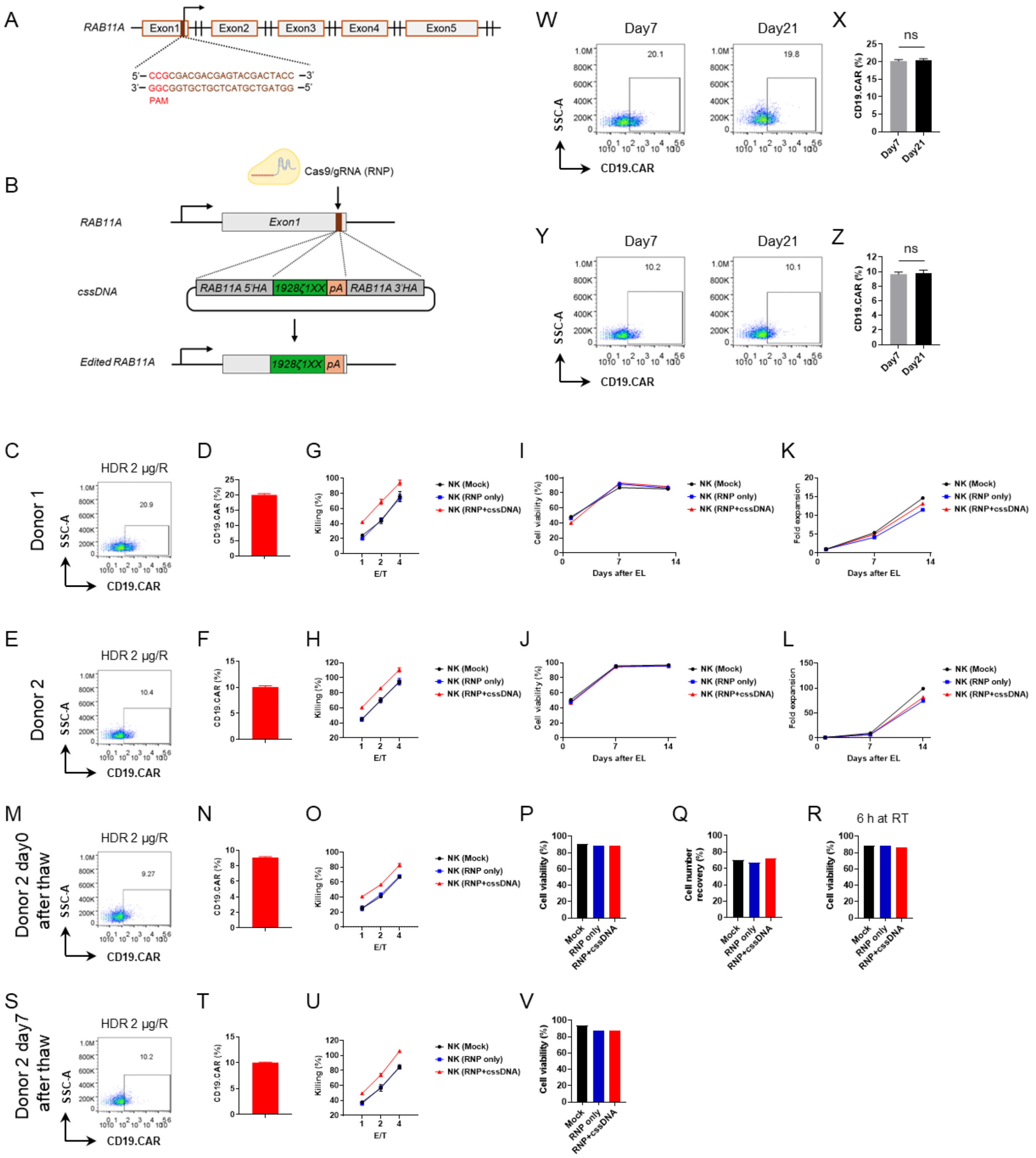

Figure S2. cssDNA donor template mediated CD19.CAR knock-in at the *RAB11A* locus.

A. sgRNA sequence targeting 3' of the exon 1 at the *RAB11A* locus.

B. Schematic diagram of using cssDNA donor template for the targeted insertion of CD19CAR (1928ζ1XX) gene at the *RAB11A* locus by CRISPR/Cas9 editing system.

C&D. Knock-in efficiency (CD19CAR %) in NK cells from donor #1 measured by flow cytometry at Day 7 post-electroporation. A representative flow cytometry profile is shown in (C), with the corresponding quantification in (D).

E&F. Knock-in efficiency (CD19CAR %) in NK cells from donor #2 measured by flow cytometry at day 7 post-electroporation. A representative flow cytometry profile is shown in (E), with the corresponding quantification in (F).

G&H. Cytotoxic activity of all NK cell groups against $CD19^{+}$ NALM6 target cells on Day 14 post-electroporation. Data are shown for donor #1 (G) and donor #2 (H).

I&J. Cell viability of all NK cell groups during 14 days of *in vitro* culture and expansion post-electroporation. Data are shown for donor #1 (I) and donor #2 (J).

K&L. Expansion of all NK cell groups during 14 days of *in vitro* culture and expansion post-electroporation. Data are shown for donor #1 (K) and donor #2 (L).

M-V. Freeze–thaw effects on NK cells. NK cells derived from donor #2, including NK (Mock), NK (RNP only), and CD19.CAR NK (RNP + cssDNA), were cryopreserved on Day 14 post-electroporation and transferred to liquid nitrogen the following day for long-term storage. After 2 weeks, cells were thawed and analyzed. On Day 0 post-thaw, knock-in efficiency (CD19CAR%) was assessed by flow cytometry (M&N), along with cytotoxic activity (O) and cell viability/recovery metrics (P–R), where P represents immediate post-thaw viability, Q represents post-thaw cell recovery rate, and R represents cell viability after 6 h at room temperature post-thaw. After 7 days of continuous culture, knock-in efficiency (CD19CAR %) was re-evaluated by flow cytometry (S&T), along with cytotoxic activity (U) and cell viability (V).

W&X. Knock-in efficiency (CD19CAR %) measured by flow cytometry analysis at two different time points (Day 7 and Day 21) post-electroporation for primary NK donor #1.

Y&Z. Knock-in efficiency (CD19CAR %) measured by flow cytometry analysis at two different time points (Day 7 and Day 21) post-electroporation for primary NK donor #2.

For D, F, G, H, N, O, T, U, X, and Z, data represent mean ± SEM of 3 independent replicates. P values in X and Z were determined using the two-tailed Student's *t*-test analysis. Herein, ns ($P>0.05$) stands for not significant.

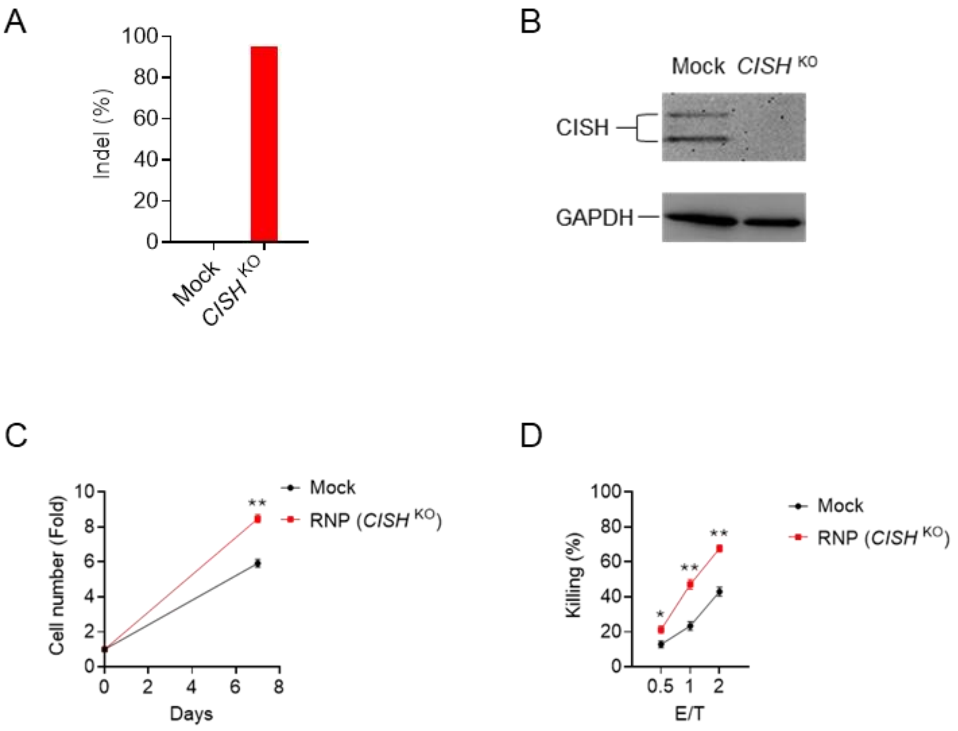

Figure S3. CRISPR/Cas9–mediated deletion of CISH in NK cells (donor #2).

A. Indel (%) at the *CISH* locus was measured by NGS amplicon sequencing.

B. Western blot assay for *CISH* protein expression in CRISPR/Cas9 engineered NK cells. GAPDH was used as loading control.

C. *In vitro* proliferation of Mock NK cells and sgRNA/Cas9 RNP complex mediated *CISH* knock-out NK cells (*CISH* ^{KO}).

D. *In vitro* cytotoxicity of Mock NK cells and *CISH* ^{KO} NK cells against K562 target cells co-cultured at various effector to target (E/T) ratios for 4 hours.

Data in C and D represent mean ± SEM of 3 independent replicates. *P< 0.05, **P< 0.01. P values were determined using the two-tailed Student's *t*-test analysis.

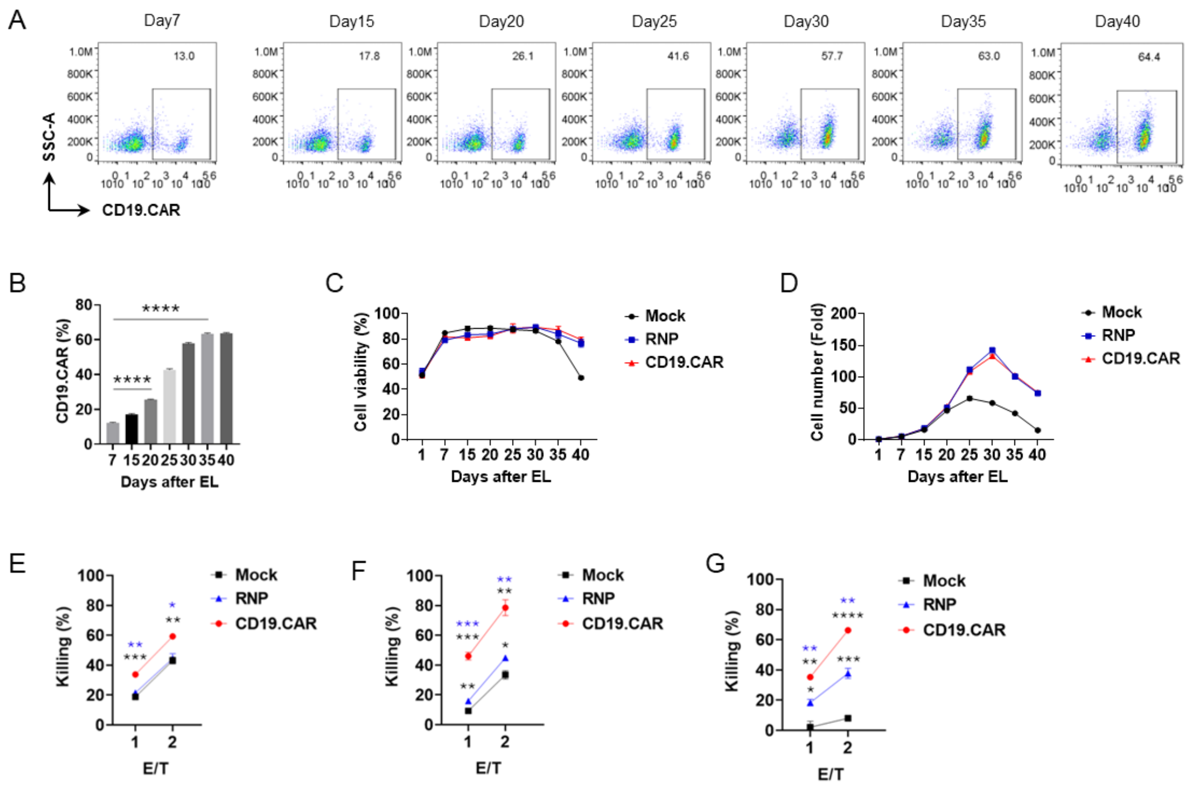

Figure S4. Efficient CD19CAR knock-in at CISH locus by CLICK in NK cells (donor #2)

A&B. Knock-in efficiency (CD19CAR %) measured by flow cytometry analysis at different time points from Day 7 to Day 40 post-electroporation. Representative FACS profiles (A) and quantifications (B). For B, data represent mean ± SEM of 3 independent replicates. ****P< 0.0001. P values were determined using one-way ANOVA analysis.

C. Cell viability analysis of all the groups of Mock, *CISH* ^{KO} and CD19CAR NK cells over the course of 40 days *in vitro* culture and expansion post-electroporation. Data represent mean ± SEM of 2 independent replicates.

D. The expansion potential of Mock, *CISH* ^{KO} and CD19CAR NK cells over the course of 40 days *in vitro* culture and expansion post-electroporation. Data represent mean ± SEM of 2 independent replicates.

E-G. *In vitro* cytotoxicity of the engineered CD19CAR NK cells against CD19 positive NALM6 target cells after co-culture at various effector to target (E/T, 1 and 2) ratios for 4 hours. Data were collected on (E) Day 7, (F) Day 21 and (G) Day 32 post-electroporation. Data represent mean ± SEM of 3 independent replicates. *P< 0.05, **P< 0.01, ***P< 0.001, ****P< 0.0001. P values were determined using one-way ANOVA analysis.

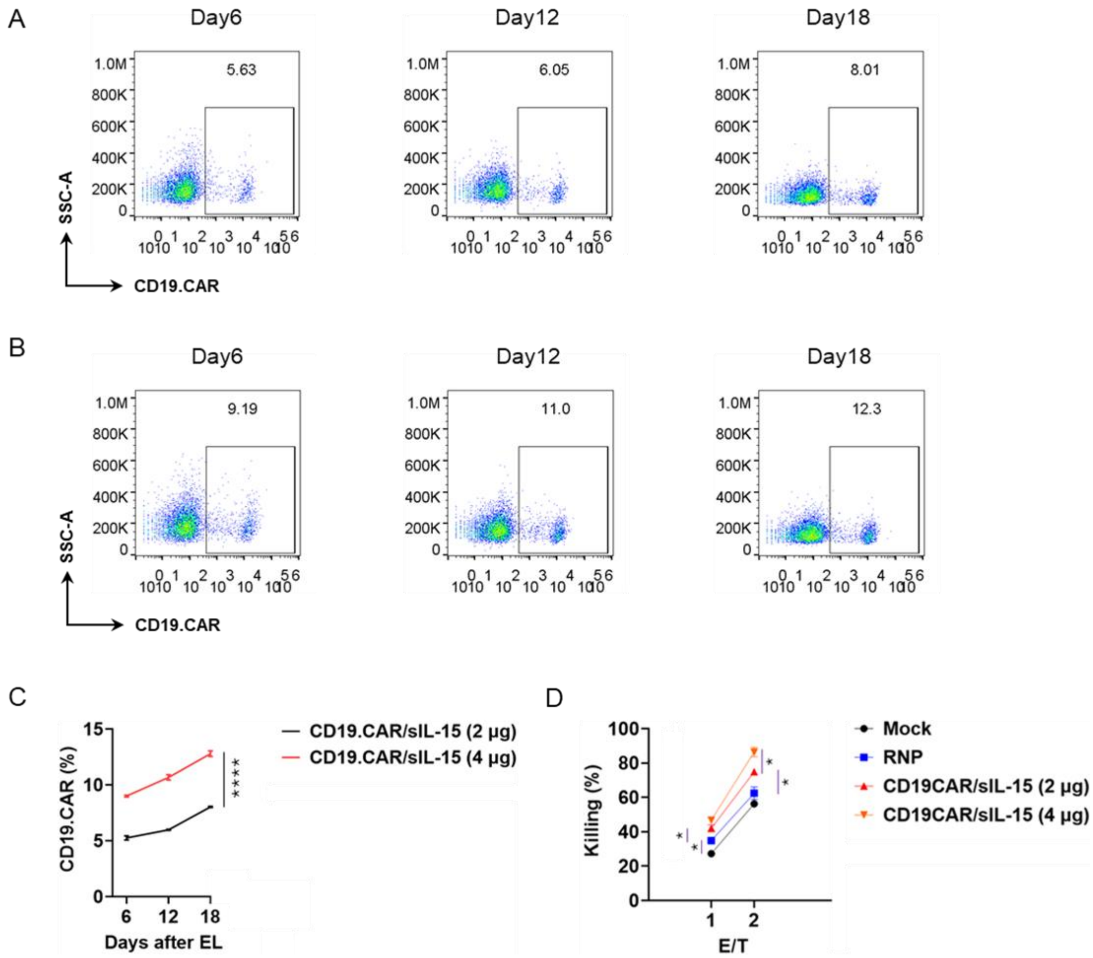

Figure S5. CD19CAR/sIL-15 CAR-NK engineering by CLICK in NK cells (donor #2)

(A-C). Knock-in efficiency (CD19CAR/sIL-15 %) measured by flow cytometry analysis at different time points from Day 7, Day 12 and Day 18 post-electroporation for donor NK cell #2. Representative FACS profiles (A&B) and quantifications (C). Here, cssDNA donor template was used for NK cell engineering at 2 and 4 μg/reaction for (A) and (B), respectively. For C, data represent mean ± SEM of 3 independent replicates. ****P< 0.0001. P values were determined using the two-tailed Student's *t*-test analysis.

D. *In vitro* cytotoxicity of the engineered CD19CAR/sIL-15 NK cells against CD19 positive NALM6 target cells after co-culture at various effector to target (E/T, 1 and 2) ratios for 4 hours. Data were collected on Day 17 post-electroporation for 2 and 4 μg of cssDNA during engineering, respectively. Data represent mean ± SEM of 3 independent replicates. *P< 0.05. P values were determined using one-way ANOVA analysis.

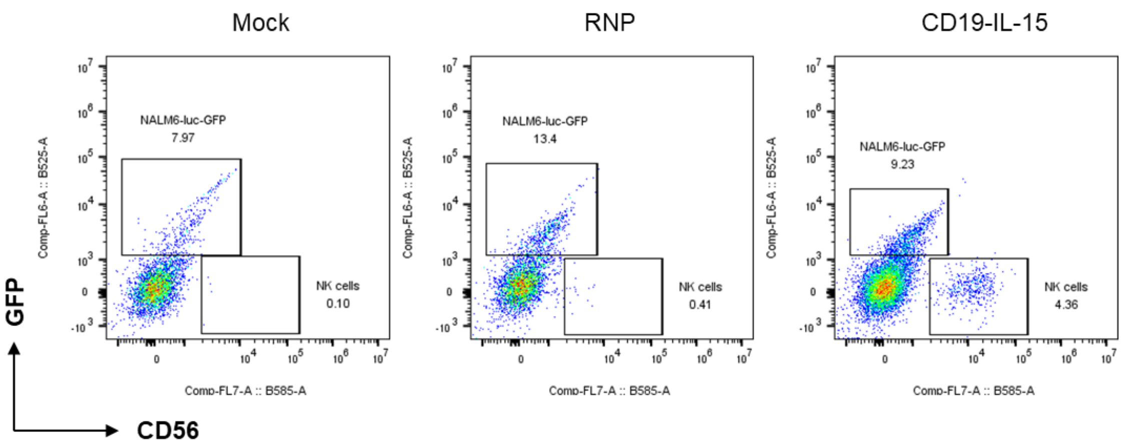

Figure S6. Characterization of CD19CAR/sIL-15 CAR-NK *in vivo* on Day 22.

FACS profiles of blood samples withdrawn from mice engrafted with Mock, RNP (*CISH* ^{KO}), and CD19-IL-15 (CD19CAR/sIL-15) NK cells, 22 days post-engraftment. GFP marked NALM-6 cells while CD56 marked adoptive NK cells.

Table S1. Information on the in-house screened sgRNAs for *CISH*

ID	gRNA sequence	PAM	Primers used for NGS	Indel (%)	Genomic location number	Note
CISH-sgRNA-1	CCAGACGGTTGATGACAAGG	CGG	F: CTCCTGCACTGCTGATACCC<br>R: GATGCCTGGAGGAGGGACA	90.94	Exon3	In-house screened
CISH-sgRNA-2	AGGCCACATAGTGCTGCACA	AGG	F: CACCTGCAGAAGATGCCAGA<br>R: AGGGCTGCACCAGTTTTAGG	56.36	Exon3	Blood. 2021;137(5):624-636.
CISH-sgRNA-3	CAAGGGCTGCATGACTGGCT	TGG	F: CTAGTCTGGGGCATAGGGGG<br>R: ACCAGATTCCCGAAGGTAGGA	40.76	Exon2	Cell Stem Cell. 2020;27(2):224-237.e6.
CISH-sgRNA-4	TGCTGGGGCCTTCCTCGAGG	AGG	F: CTAGTCTGGGGCATAGGGGG<br>R: ACCAGATTCCCGAAGGTAGGA	42.06	Exon2	Cell Stem Cell. 2020;27(2):224-237.e6.
